# Drilling and blasting designs for parallel hole cut and V-cut method in excavation of underground coal mine galleries

**DOI:** 10.1038/s41598-023-29803-6

**Published:** 2023-02-11

**Authors:** Ozgur Yilmaz

**Affiliations:** Department of Mining and Mineral Extraction, Zonguldak Vocational School, Zonguldak Bülent Ecevit University, 67500 Zonguldak, Turkey

**Keywords:** Energy science and technology, Engineering

## Abstract

The Karadon mine, where field studies were carried out, is located in the north west of Turkey and is considered to be a highly gaseous coal mine. Explosives and ignition systems used in underground coal mines are determined by laws, statutes, regulations and strict rules. Restrictions arising from legal requirements, such as restrictions on charging and stemming lengths and blasting agents to be used, make the use of well-known tunnel blasting techniques difficult or sometimes not possible. In such cases, the designs made with the equations given in the literature must be revised and rearranged. The objective of this study is to recommend solutions to the blasting difficulties that are encountered in gaseous underground coal mines in which there are limitations arising from legal requirements. This study summarizes and analyses the blasting practices currently employed at Karadon mine along with their disadvantages. New blasting designs were then made using the methods suggested in the literature, and these designs were revised according to legal requirements. By keeping the charge concentration constant relative to its original value, the burden and spacing distances were adjusted. As a result of this study, it has been seen that rearrangement by keeping the charge concentration constant compared to its original value is an appropriate engineering solution.

## Introduction

Explosives are probably the most cost-effective method of rock excavation in underground mining operations^1–3^. Tunnel and underground rock excavation have largely relied on drilling and blasting methods in recent years^4^. Quantity and type of explosives, drilling patterns, and the sequence of initiation are all critical to the successful excavation of rock masses^5^.

Blasting in a tunnel or drift always begins with the "cut" -a pattern of holes designed to provide the most efficient line of deformation- at or near the centre of the face. Each blast creates more space for the following ring of blast holes. It is imperative to blast the cut section successfully for the whole round to be successful. In mining and construction, various cut types have been applied, but they fall essentially into two categories: parallel holes and holes drilled at angles. Today, the most commonly used parallel hole cut method is the four-section large hole cut, whereas the most commonly used cut with angled holes is the V-cut. This type of cut is effective for tunnels with a fairly large cross-section and requires fewer holes than a parallel cut. When the first mechanized drilling machines were introduced, the parallel cut enabled accurate parallel drilling. The use of this type of cut is also common in small tunnels with long rounds.

The type of cut chosen depends not only on the physical nature of the rock and the presence of weakness and crack sets, but also it depends on the equipment used, the cross-sectional area and the preferred advance rate. Over the years since explosives were first used to excavate tunnels in the 1860s, tunnel blasting has gained enormous experience. Many researchers^6–13^ examined cut blasting and proposed a variety of cut patterns. As far as cutting methods are concerned, Shapiro^14^ compared V-cuts to other cuts and concluded that the V-cut provides the maximum blasting efficiency when the hole is less than 2.5 m deep. Chakraborty et al.^15^ showed that parallel cuts were not as productive as V-cuts in small tunnels, and an empirical relationship was developed. In different blasting models, Soroush et al.^16^ investigated the effect and sensitivity of hole diameter and tunnel face area on blasting results and pointed out that V-cut requires more cut holes than parallel cut under similar conditions. Cardu and Seccatore^17^ report that tunnel rounds with parallel hole cuts tend to have a higher pull efficiency than tunnel rounds with inclined hole cuts. According to Wang et al.^18^, V-cuts have the advantages of fewer boreholes, easy rock casting, and low drilling accuracy requirements. However, the trend toward parallel hole cuts has emerged as a result of the development of hydraulic jumbos with one or more booms. Moreover, parallel hole cuts do not require a change in feed angle and advance is not affected by tunnel width as much^19^.

The lack of a free surface makes cut blasting more difficult than open-pit blasting^20^. Assuming that the bottom, left, and right sides of the cut rock mass were broken by explosion, and that shear failure occurred on the upper and lower sides, the failure modes of the different sides of the cut rock mass were fully considered by Dai and Du^21^. The mechanism of cavity formation in V-cuts was qualitatively explained by them. A calculation method for determining the critical depth of the central hole was proposed by Lou et al.^22^ based on his theory of the V-cut blasting mechanism.

The existence of empty holes can create favourable crushing conditions for rock during the blasting process of straight cuts. Therefore, the primary way to increase the blasting effect of mine tunnelling is to optimize and improve the cutting method^23^. Based on their study of cavity formation in gutters, Lin and Chen^24^ concluded that rock would be thrown out under the action of gas generated by a high-pressure explosion. Field tests were conducted by Liu et al.^25^ to determine how the central hole affects stress concentration. According to the above-mentioned researchers, the hole boosts cyclic advancement by enhancing rock fragmentation via reflection of tensile waves. In order to combine the advantages of parallel and angled cutting, Shan et al.^20^ invented quasi-parallel cutting with a centre hole. Furthermore, Shan et al.^20^ proposed an angle between the main cut and the working face, while the auxiliary cut was perpendicular. By using a central hole, Yang et al.^26^ demonstrated that the throwing effect of broken rocks and the utilization of cut holes could be greatly improved. Wang et al.^4^ conducted numerical simulations to analyse the explosive stress field generated by the first-order cut hole. According to the same authors, the increase in the diameter of an empty hole made the stress wave peak value around the hole more than twice as high as in a rock mass with no empty hole. Zuo et al.^27^ observed the blasting process at different hole diameters using high-speed photography and explained the relationship between cavity volume and hole diameter using high-speed photography.

The above researches are primarily focused on the cutting mechanism and cutting method, but the researches on gallery blasting in gaseous underground coal mines are not sufficiently mentioned. As a result of mining, methane is released from coal and the surrounding rock strata. It is extremely dangerous to mix methane with air in mines because of its explosive properties. For this reason, explosives and ignition systems used in gaseous underground coal mines are determined by laws, statutes, regulations and strict rules. These legal requirements, which restrict charging and stemming lengths, blasting agents to be used and the use of delayed capsules, make the use of well-known tunnel blasting techniques difficult and sometimes impossible. In this case, the blastholes cannot be drilled to the required length or cannot be charged with sufficient explosives. Due to this, the required linear charge concentration cannot be achieved. Additionally, when the delayed capsules are not used, each blasthole group such as cut, stoping and perimeter have to be blasted at the same time. It usually takes two or three steps to drill and blast (charging and ignition) blastholes when delayed detonators are not used. In this case, rocks become more fixed and more difficult to break and evacuate from the face. Also hard rock blasting is very challenging with methane safe permissible dynamites due to their low relative weight strength. Consequently, such difficulties result in insufficient blasting or insufficient advancement in excavation, especially in gaseous hard coal mines. In such cases, it is unclear how tunnel blasting techniques recommended in the literature produce a solution that complies with these limitations, especially in gaseous underground hard coal mines. There are no formulas given in the literature that can be used directly in such special cases. To overcome these difficulties, designs made with the equations given in the literature must be revised and rearranged considering specific charge concentrations.

In the Turkish Hard Coal Enterprise (TTK) which is the largest hard coal producer in Turkey, large galleries such as development roadways (excavated in rock) are blasted by parallel hole cut method whereas short and narrow galleries such as gateroads and drifts (partially in or near the coal seam) are blasted by V-cut method. In this study, the blasting practices currently employed at Karadon mine are summarized and the current situation is presented with its disadvantages. Then, firstly, new blasting designs that would increase the current advance were made using the methods suggested in the literature, and then these designs were revised considering the legal requirements. By keeping the charge concentration constant relative to its original value, the burden and spacing distances were adjusted. Parallel hole cut designs were made for development roadways, whereas angled hole cut designs were made for gateroads and drifts. The drilling pattern, the numbers and amounts of the required delayed blasting caps and the amount of dynamites were provided in this context.

## Design requirements in Turkey for blasting in coal mining

The use of explosives and ignition systems in underground coal mines is governed by laws, statutes, and regulations. There are two specific laws in Turkey which clearly outline the use of explosives in mines and occupational safety. The first legislation is the requirements set forth in the decree no 87/12028 "Regulations/Directives about Procedures and Principles for Hunting Materials and Similar Items related with Non Monopoly Explosive Articles" and the other one is the "Regulation on OHS to be taken in the Mining and Quarrying Operations and Tunnel Construction"^28^. In these two legislations, the articles including drilling and blasting applications specific to underground coal mining are included only in the first one. Summary of these articles related to tunnelling in coal mines is provided as in the following:

Article 14—: In the construction of mines, quarries and tunnels only blasting agents of the type permitted by the Ministry can be used. The blasting agents to be used gaseous, coal dust mines and sulphur mines must be of the quality required by the safety of these mines.
Article 26—: The height of the explosive charge cannot exceed half the hole depth. The remaining space is filled with stemming material.
Article 27—: In mines with flammable and combustible gases and danger of dust burning and explosion, firing with fuse cannot be made
Article 35—: Fire-proof electrical ignition device will be used in gaseous and coal dust mines.
Article 38—: c) Delay capsules can only be used in coal mines for blasting in rock;
  d) If sudden gas release in coal mines is suspected or if gateroads are approaching the coal seam, safe capsules are used instead of delayed ones.
Article 39—: a) In coal mines, gateroads can only be ignited by electric capsules.
  b) Capsules with aluminium shells cannot be used in coal and sulphur mines.

Drilling and blasting in coal mines are difficult to design due to the above-mentioned requirements.

## Site description and encountered rock formations

**Figure 1:**
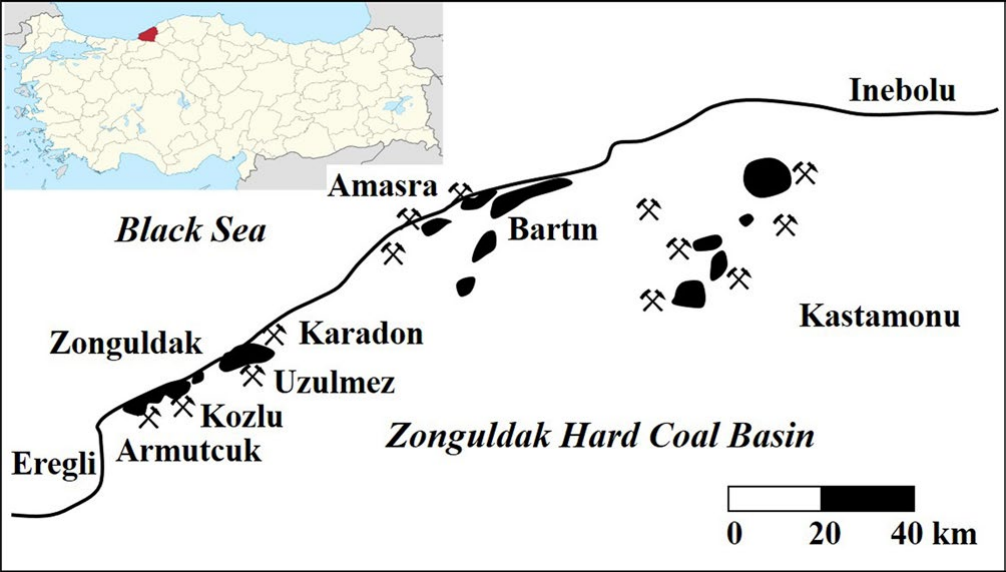
Location of the mine collieries in the Zonguldak hard coal basin.

The main hard coal deposits in Turkey are located in the Zonguldak basin, between Ereğli and Amasra on the Black Sea coast, with a distribution of 160 km. As the largest producer of hard coal in Turkey, TTK manages all hard coal activities in the country as a state-owned enterprise. Due to the dispersed nature of the Zonguldak hard coal basins, TTK has established five production areas: Armutcuk, Kozlu, Uzulmez, Karadon, and Amasra. The first four of the establishments are within the borders of Zonguldak province and the last one within the province of Bartın (Fig. 1).Underground mining is used for all of the production, and extraction occurs simultaneously at several horizons within the collieries. Coal seams in the coalfield have an average thickness of 2 m and an inclination of 0°–90°. The calorific value of the Zonguldak basin coals ranges between 6200 and 7250 kcal/kg and the estimated reserve is approximately 1.3 billion tons.

There are more than 20 mineable coal seams in the Zonguldak coalfield, which consists of a belt of carboniferous coal measures. The geology is characterized by steeply dipping seams which in certain areas lie beneath the Black Sea. Faults and folds have significantly disturbed the strata, and methane is present in large quantities. Besides its tendency toward spontaneous combustion, coal is also known to be prone to outbursts. As a result of these problems, mining has developed into a highly labour-intensive industry, with limited potential for mechanization. The Karadon Hard Coal Business Enterprise (Karadon TIM), where field studies were carried out, continues its production activities in an area of 32 km^2^, 12 km east of Zonguldak province and is categorized as a highly gaseous coal mine. As a consequence of mining activities, coal mine methane is released from coal and surrounding rock strata. Mine methane poses a safety risk due to its explosive nature.

**Figure 2:**
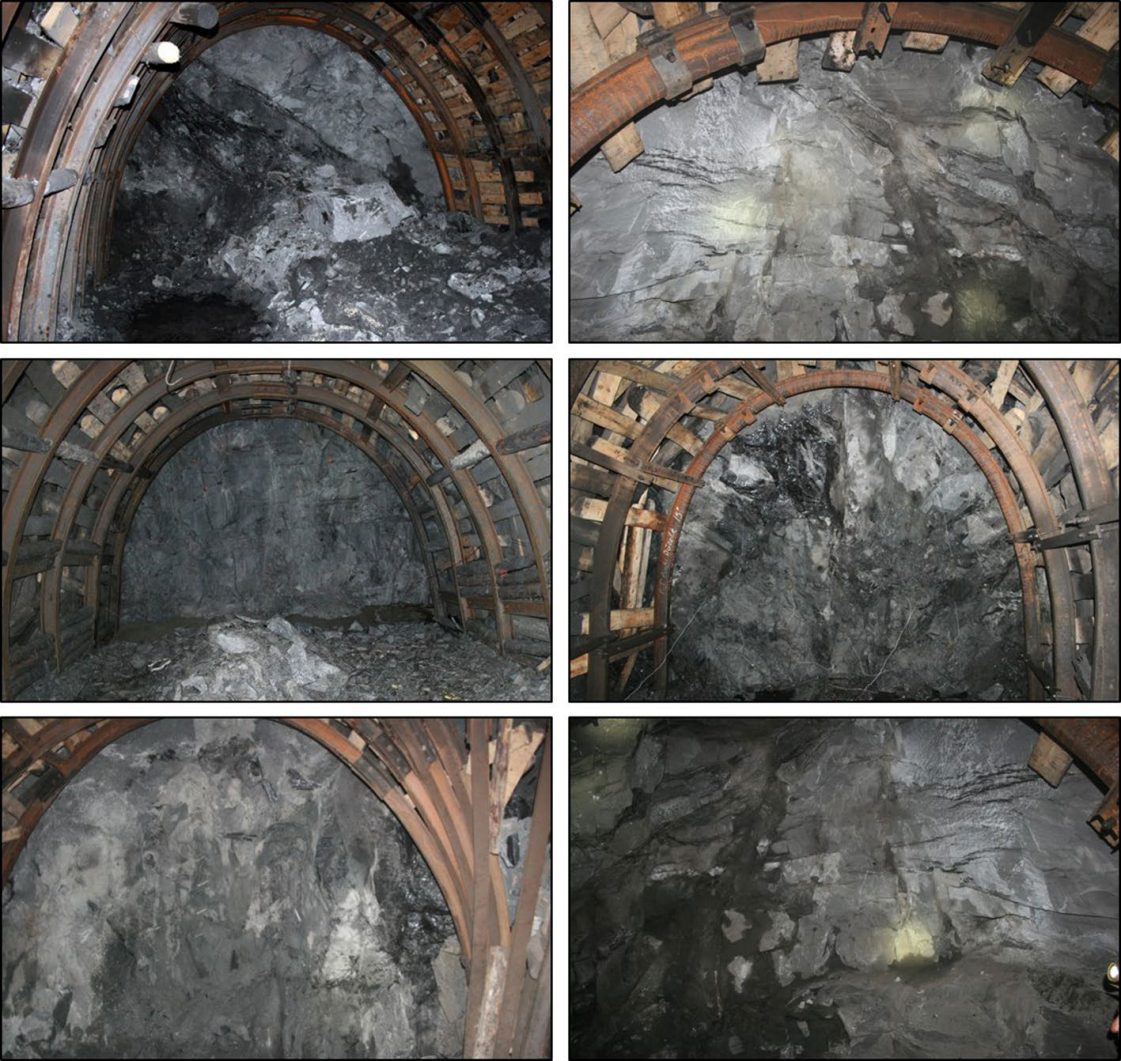
Observed rock formations in the coal basin.

In order to determine the elastic and mechanical properties of the surrounding rock, rock mechanics experiments were conducted on 15 different mineable coal seams by sampling the floor and roof strata. The surrounding rock is mainly composed of sandstone, siltstone and conglomerate (Fig. 2). The summary of the average values of the intact rock properties obtained from the tested rocks is presented in Table 1.

**Table 1:** Intact rock properties of frequently encountered rock formations.

Intact rock properties	Sandstone	Siltstone	Conglomerate
Uniaxial compressive strength (σ_c_) (MPa)	74.5 ± 29.7	30.8 ± 17.1	57.8 ± 14.9
Brazilian tensile strength (σ_tB_) (MPa)	6.10 ± 2.84	4.61 ± 2.20	3.15 ± 1.77
Deformation modulus (E) (GPa)	32.6	29.1	24.8
Poisson’s ratio (υ)	0.22	0.22	0.24
Cohesion (c) (MPa)	24.1	16.7	9.4
Internal friction angle (ϕ) (^o^)	40.0	36.3	46.0

**Figure 3:**
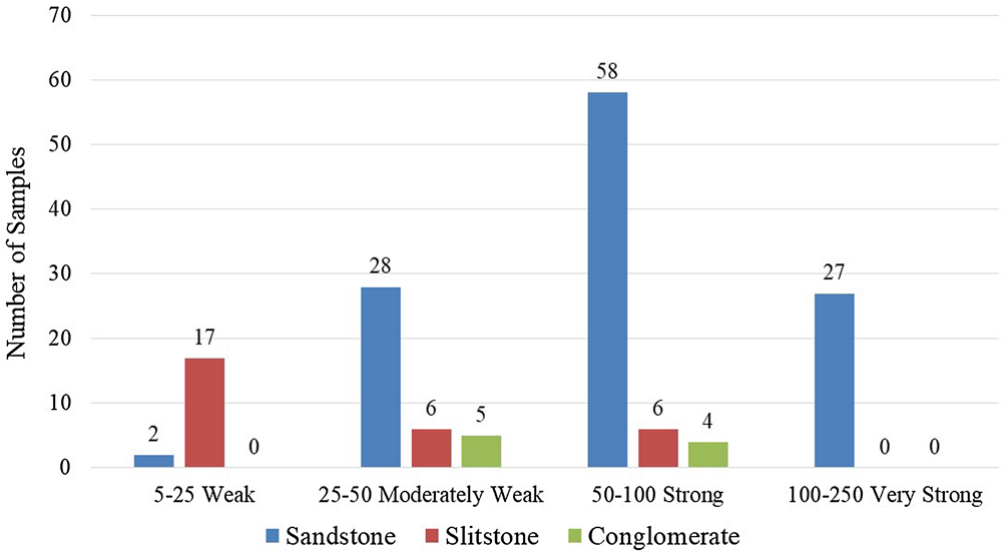
Classification of the uniaxial compressive strength of tested rocks.

According to the classification suggested by ISRM^29^ in terms of uniaxial compressive strength of rock materials, the distribution of tested rock samples is shown in Fig. 3. In general, the UCS (Uniaxial Compressive Strength) values of the sandstone samples were classified as strong from an engineering perspective, while the siltstone samples were classified as weak and the conglomerate samples were classified as moderately weak. However, 27 of the samples of sandstone had UCS values higher than 100 MPa with a maximum value of 140 MPa. This value remains in the 100–250 range of the ISRM recommended classification and is designated as very strong rocks.

Based on experience from the mine, the formations encountered in the field are divided into categories according to rock strength for simplicity. These categories include medium hard formations and relatively weak formations. The rock mass characteristics in the region are variable and the average RQD (Rock Quality Designation) is around 90% in medium hard formations which contain massive sandstone while this value is around 20% in relatively weak formations that consist of siltstone-sandstone. RMR (Rock Mass Rating) values are around 70 in medium hard formations with massive sandstone, and 40 to 60 for relatively weak formations with weak siltstone, sandstone, and conglomerate transition units.

## Tunnel blasting by drilling and blasting

Process of excavating tunnels, galleries, drifts, etc. are called tunnelling. Drilling and blasting are a widely used excavation method in tunnelling applications due to its versatility, lower initial investment cost and easier applicability compared to mechanical excavation. Although tunnelling machines are used in many transportation tunnel projects today, by far the most common technique of underground rock excavation is that of drilling and blasting.

Bench blasting differs from tunnel blasting in that bench blasting is directed toward multiple free surfaces whereas tunnel blasting is directed toward a single free surface. As a result, there is a limit on the round length and volume of rock that can be blasted simultaneously. In order for the rock to break and be thrown away from the surface, a second free face must be formed. A cut in the tunnel face produces this second face^7^. The purpose of the cut is to create a primary cavity in the tunnel line. This is where the remaining holes can easily expand and blast the remaining rock towards this opening.

Mining and construction use a variety of cut types, but they fall mainly into two categories: parallel-hole cuts and angle-hole cuts. Although there are many different subtypes of each major cut type, the most common types of cut today are parallel hole and V-cut.

### Parallel hole cut

With the introduction of mechanized drilling machines, parallel hole cut was introduced^11^. All holes are drilled parallel to each other in this type of cut. A large drill hole is used as an opening for blasting. The most used parallel hole cut method today is the four-section large hole cut, which is also known as the Swedish method. Originally developed by Langerfors and Kihlstrom^6^, a complete design model was later published by Holmberg^9^ for this cut design. Recently, it was updated by Persson et al.^13^.

In parallel hole cut, the advance of the round is restricted by the diameter of the empty hole and the deviation of the charge holes. Since drifting will be quite expensive if the advance is much less than 95% of the drilled hole depth, the average advance (I) can reach 95% of the blasthole depth (H).

1 $${\text{I}} = 0.{\text{95H}}$$

**Figure 4:**
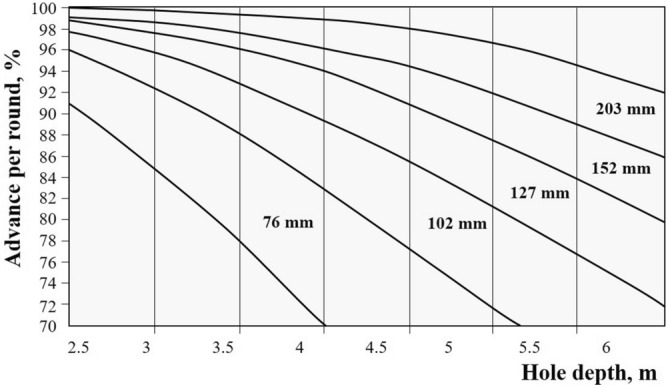
The relationship between hole depth and advance for different empty hole diameters^10^.

In four-section parallel hole cuts, the depth of the blasthole can be estimated by the following equation;

2 $$\mathrm{H}=0.15+34.1{\mathrm{D}}_{0}-39.4 {\mathrm{D}}_{0}^{2} (0.05 \le \mathrm{ D}0\le 0.25\mathrm{ m})$$

where D_0_ denotes the empty hole diameter (m)^13^. Olofsson^10^ also proposed the chart shown in Fig. 4 for the relationship between hole depth and advance for different empty hole diameters.

The breakage mechanism is greatly dependent upon the type of explosive, the rock properties and the distance between the empty hole and the charged blastholes^12^. For the overall blasting procedure, determining the burden of cut holes is crucial^30^. For an effective breakage process, the distance between the empty hole and the blasthole in the first quadrangle B_1_ should not exceed 1.7 times the diameter of the empty hole D_0_^6^. For burdens larger than 2D_0_, the break angle is too small and a plastic deformation of rock between the blastholes is produced. If the rock burden is under D_0_, cut failure will occur. For this reason it is recommended that the burdens to be calculated as B_1_ = 1.5D_0_. According to Persson et al.^13^, if the drilling deviation is more than 1%, the burden in the first quadrangle is practically calculated as follows;

3 $${\mathrm{B}}_{1}=1.7{\mathrm{D}}_{0}-\left({\mathrm{\alpha }}_{e}\mathrm{H}+{\upbeta }_{e}\right)$$

where the term $\left({\mathrm{\alpha }}_{e}\mathrm{H}+{\upbeta }_{e}\right)$ represents the maximum drill deviations, α_e_ is the angular deviation in m/m. H is the hole depth in meters, and β_e_ denotes the collaring deviation in meters. The linear charge concentration q (expressed in kg/m), is calculated from the following equation^13^;

4 $$\mathrm{q}=55 {\mathrm{d}}_{h}{\left(\frac{\mathrm{B}}{{\mathrm{D}}_{0}}\right)}^{1.5}\left(\mathrm{B}-\frac{{\mathrm{D}}_{0}}{2}\right)\left(\frac{\mathrm{c}}{0.4}\right)\frac{1}{\mathrm{RWS}}$$

where the term d_h_ denotes the blasthole diameter (m), D_0_ indicates the diameter of the empty hole (m), B is defined as the maximum distance between holes and burden (m), c is the rock constant and RWS is expressed as the relative weight strength of the explosive with respect to ANFO. Assuming that the burden in the first quadrangle (B_1_) are known, the following formula is used for the geometric design of width of first quadrangle^10^.

5 $${\mathrm{W}}_{1}={\mathrm{B}}_{1}\sqrt{2}$$

After the first quadrangle is calculated, the burden for the following quadrangles should be solved geometrically. Blasting toward a circular hole demands a higher charge concentration than blasting toward a rectangular opening. This occurs due to higher and less effective stress wave reflection. The calculation method for the remaining quadrangles of the cut is essentially the same as for the 1^st^ quadrangle. The breakage, however, occurs towards a rectangular opening instead of a circular one. There are many well-tested variants of the parallel hole cut patterns that function well^11^. According to Langerfors and Kihlstrom^6^, the burden (B) for the remaining quadrangles of the cut should be 0.7 times the width (W) of the existing opening in order to provide maximum breakage (B_n_ = 0.7W_n_). Then, the width (W_n_) of the remaining quadrangles can be calculated geometrically as in the following^8^;

6 $${\mathrm{W}}_{n+1}=1.7{\mathrm{W}}_{n}$$

**Figure 5:**
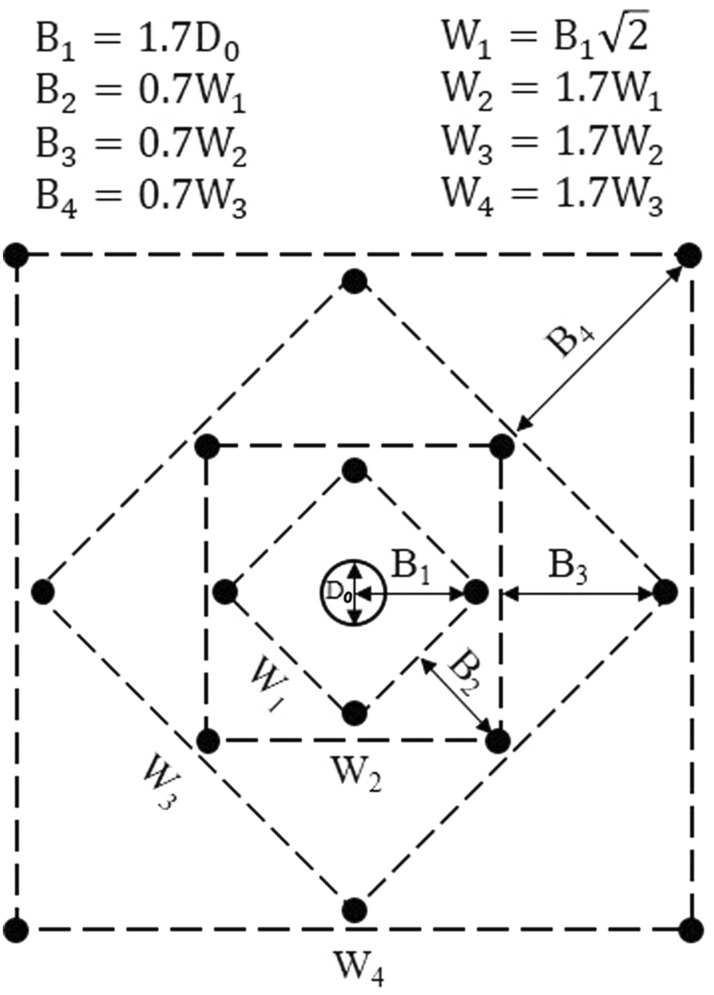
Four-section large hole cut design.

The four-section large hole cut design dimensions are shown in Fig. 5. As a common rule of thumb for determining the number of section, the side length of the last section should not be less than the square root of the advance^12^. The stemming length (h_o_) should be estimated as 10 times the blasthole diameter.

**Figure 6:**
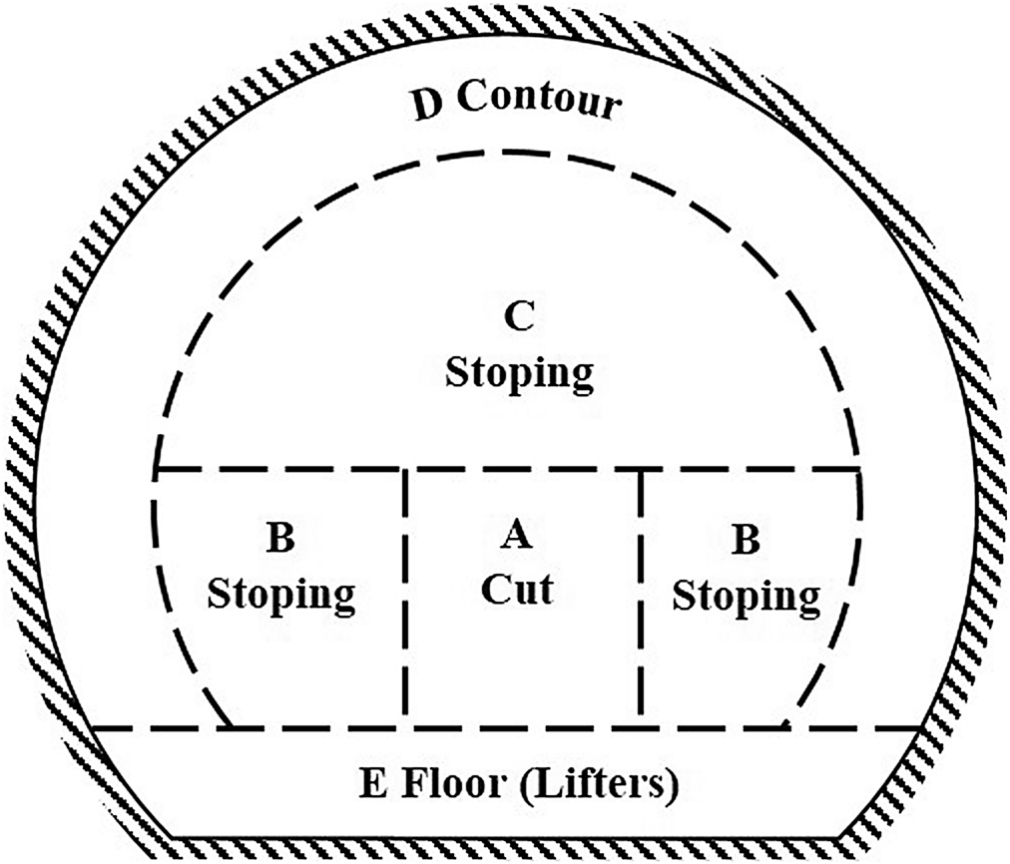
Zones in tunnel blasting^9^.

When the cut holes (A) have been calculated, the rest of the tunnel round may be calculated. The round is divided into: stoping holes with breakage upward and horizontally (B), stoping holes with breakage downwards (C), contour holes (roof and wall holes) (D) and floor holes (lifters) (E)^10^. Figure 6 illustrates the different tunnel segments created by tunnel blasting. The burden (B) for the round depends on the amount of explosive and the diameter of the blasthole. The burden equation is presented as follows;

7 $$\mathrm{B}=30{\mathrm{q}}_{\mathrm{b}}/{\mathrm{d}}_{h}$$

where, the term B represents the burden (m), d_h_ denotes the blast blasthole diameter (mm) and q_b_ is the bottom charge concentration (kg/m). Calculation of bottom charge concentration is as in the following^10^;

8 $${\mathrm{q}}_{\mathrm{b}}=\frac{\uppi {d}_{h}^{2}}{4}{\mathrm{q}}_{e}\mathrm{ AWS}$$

where, q_e_ is the explosive density (g/cm^3^) and AWS is absolute weight strength (1 for Dynamex M)^31^. When the burden (B), the hole depth (H) and the bottom charge concentration (q_b_) are known, Table 2 can be used to calculate the drilling and charging geometry of the round.

**Table 2:** Drilling and charging geometry for parallel hole cut^8,10^.

Part of the round	Burden, B (m)	Spacing, E (m)	Height of bottom charge, h_d_ (m)	Charge concentration		Stemming (m)
				Bottom, q_b_ (kg/m)	Column, q_c_ (kg/m)	
Stoping holes						
Upward and horizontally	B	1.1 × B	H/3	q_b_	0.5 × q_d_	0.5 × B
Downwards	B	1.2 × B	H/3	q_b_	0.5 × q_d_	0.5 × B
Roof holes	0.9 × B	1.1 × B	H/6	q_b_	0.3 × q_d_	0.5 × B
Wall holes	0.9 × B	1.1 × B	H/6	q_b_	0.4 × q_d_	0.5 × B
Floor holes	B	1.1 × B	H/3	q_b_	1 × q_d_	0.2 × B

### Cut with angled holes

The most commonly used cut with angled holes is the V-cut. Using symmetrically drilled angled holes, the V-cut is a traditional cut. The lower middle of the face is usually pierced with wedge-shaped angled holes. The first detonation will remove the material in the wedge, allowing subsequent detonations to break the wedge^32^. In wide tunnels where drilling is not limited by tunnel width, it is still commonly used.

**Figure 7:**
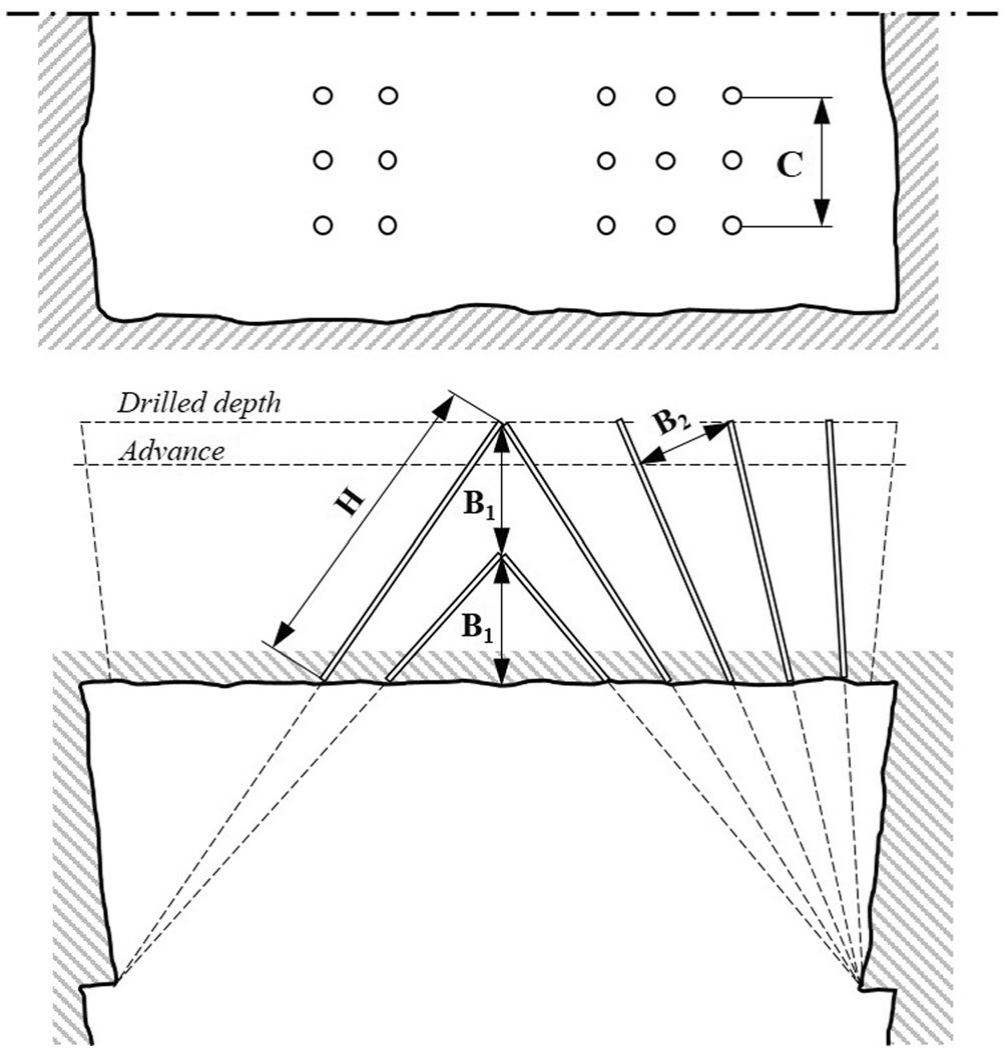
Typical V-cut used in underground mining^10^.

V-cuts require a certain tunnel width in order to accommodate drilling equipment (Fig. 7). Therefore, the tunnel width greatly limits the use of the V-cut. In this type of cut design, the theoretical advance per round increases with the tunnel width and an advance of 45–50% of the tunnel width can be achieved. In the case of narrow tunnels, the V-cut becomes pointed and thereby more difficult to blast. As a result, small tunnel excavation requires more rounds of drilling. Drilling precision influences the results of blasting to a significant extent. Normally the cut consists of one or two V’s, but in deeper rounds, it may consist of triple or quadruple V’s. Surface blasting principles are used for V-cutting in which the angle of rock expansion equals or exceeds 90 degrees. The angle of cut must not be too acute and should not be less than 60°. More acute angles require higher charge concentrations in the holes^8,10^.

**Figure 8:**
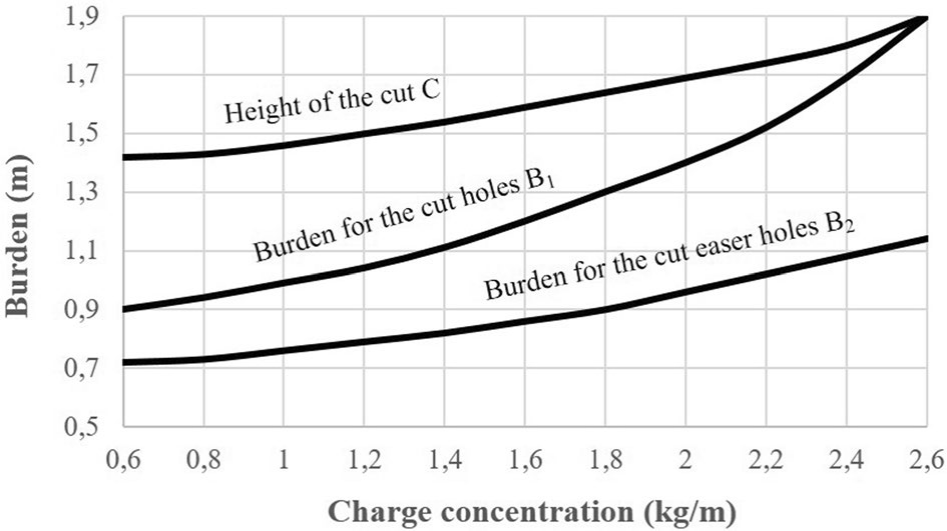
The burdens B_1_, B_2_ and the cut height C in relation to the bottom charge^10^.

Tunnel excavation using V-cut blasting will result in a minimum burden increase as depth increases^22^. Each V’s burden depends on the amount of explosive with which the blasthole diameter can be charged. In the Swedish model the equation that gives the burden, depends mainly on linear charge concentration (drillhole diameter) and type of explosive. The relation that gives the burden is;

9 $$\mathrm{B}=30{\mathrm{q}}_{\mathrm{b}}.\mathrm{f}/{\mathrm{d}}_{h}$$

where, the term B represents the burden (m), d_h_ denotes the blast blasthole diameter (mm), f is the burden correction coefficient according to angle of cut holes α_v_ (f = 1 for α_v_ = 60°; f = 1.1 for α_v_ = 75°; f = 1.2 for α_v_ = 90° and the values in-between can be interpolated) and q_b_ is the bottom charge concentration (kg/m) and can be calculated from Eq. (8). The spacing (E) for the cut holes should be 0.8 times the burden (B). The bottom charge should be at least one-third of the depth of the hole. The column charge concentration should be 0.5 times the bottom charge. The uncharged part of the hole is 0.3 times of the burden. Figure 8 gives the height of the cut (C) and the burdens B_1_ and B_2_ for the cut in accordance with the charge concentration^10^.

For the rest of the round, the method of calculation is the same as presented in Table 2. Cut spreading holes must be detonated to the greatest possible extent by the use of millisecond detonators.

## Drilling and blasting round designs at the application site

Physical and chemical examinations and gallery tests must be carried out to determine whether the explosives used in underground coal mines are safe against fire and coal dust. Before using any type of explosives, it is absolutely necessary to obtain the permission of the regulatory agency. Two types of dynamite are used in Karadon TIM as explosives, namely methane safe permissible dynamite and gelatine dynamite. Methane safe permissible dynamite is primarily suitable for underground coal blasting operations and for the blasting of soft rocks. Hard rock blasting cannot be accomplished with this type of dynamite due to its low relative weight strength. Additionally, it is the only reliable product tested in underground coal mines in Turkey for mine gases (particularly methane) and coal dust^33^. Nevertheless, the company is allowed to use a certain type of gelatine dynamite in galleries driven into rock (at least 20 m distance from coal seams) provided that it complies with the legal requirements previously mentioned. The technical properties of methane safe permissible type dynamite and gelatine dynamite are listed in Table 3. This mine also uses electric-delay blasting caps (copper) according to safety regulations.

**Table 3:** Technical properties of methane safe permissible and gelatine type dynamites^33^.

Parameters	Values	
	Methane safe dynamite	Gelatine dynamite
Density (g/cm^3^)	1.10	1.50
Cartridge length (mm)	200	200
Detonation velocity (m/s)	3500	7500
Absolute weight strength, AWS	0.56	0.80
Relative weight strength, RWS	0.655	1.20
Linear charge concentration, q (kg/m)	0.625	1.20

### Parallel hole cut designs (development roadways)

The development roadways (opened in rock formations) in Karadon TIM are excavated with gelatine dynamite using the parallel-hole cut method. In this method, blasting is done in two stages; first the cut and cut spreader holes are blasted, then the rest of the roadway face is drilled and blasted (stoping, contour, and lifter holes). The empty hole is first bored 38 mm and then gradually expanded to 76 or 115 mm, depending on rock strength.

The nominal diameter and length of the used drill at the mine are 0.038 m and 3 m, respectively. For the electro-hydraulic drilling equipment used, the angular and collaring deviations were estimated as 0.2 m/m and 0.2 m, respectively. 2.4 m of advance is provided for a 14 m^2^ cross-section area with 90 blastholes. Based on experience from the mine, the diameter of the empty hole should be 115 mm in medium hard formations and 76 mm in relatively weak formations. Therefore, two different blasting designs were considered according to rock strength.

The length of the used drill at the mine is 3 m, so it would be appropriate to take the actual hole depth as 2.85 m (H = 2.85 m). In this case, the average advance can be achieved in one round with 95% efficiency, which corresponds to 0.95 H = 2.7 m. Practical burden can then be calculated from Eq. 3 for both empty hole diameters;

10 $${B}_{1(76)}=1.7\cdot 0.076-\left(0.02\cdot 2.85+0.02\right)=0.05 \mathrm{m} \mathrm{for }76\mathrm{ mm empty hole diameter}$$

11 $${B}_{1(115)}=1.7\cdot 0.115-\left(0.02\cdot 2.85+0.02\right)=0.12 \mathrm{m for }115\mathrm{ mm empty hole diameter}$$

In this work, the rock constant c was chosen as 0.4 kg/m^3^ for massive sandstone formations and 0.45 kg/m^3^ for relatively weak siltstone, sandstone and conglomerate transition formations. Thus, using Eq. 4, the linear charge concentrations are calculated as 0.386 and 0.520 kg/m for 76 and 155 mm empty hole diameters, respectively.

As can be seen, the linear charge concentration of gelatine dynamite (1.20 kg/m) used exceeds the required linear charge concentration in the blasthole. This may cause rock residues left at the hole bottom after blasting to bulge and even cause cutting failure. To prevent this phenomenon, it is necessary to calculate the distance between the empty hole and the blasthole in the first quadrangle according to the current dynamite features. These calculations can be made by trial and error method until reaching the required linear charge concentration of gelatine dynamite. In this manner, the burden distance is adjusted so that the charge concentration of the dynamite used will provide the required charge concentration. When these calculations are made, the practical burdens are obtained as 0.113 and 0.182 mm for 76 and 155 mm empty hole diameters, respectively.

**Figure 9:**
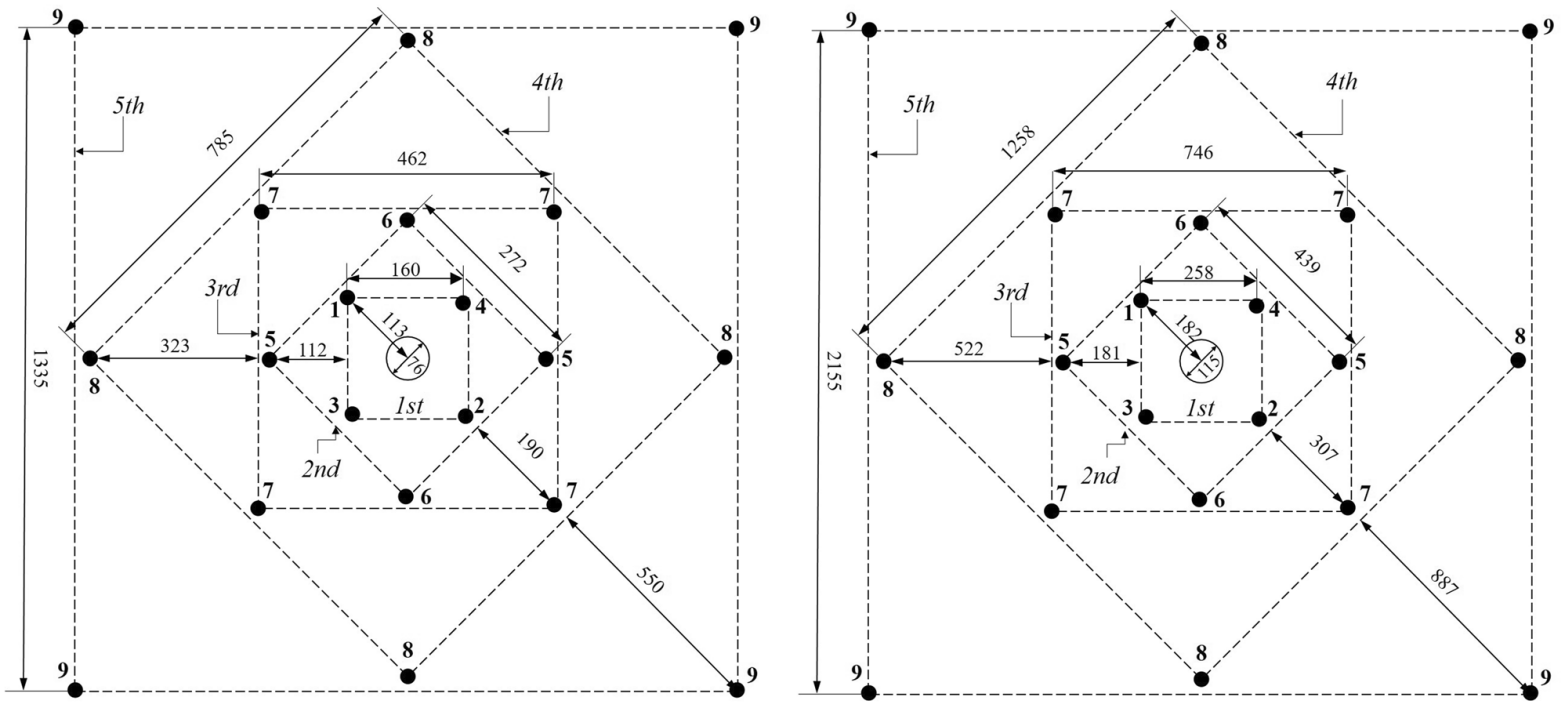
The arrangement of cut holes for 76 and 115 mm empty hole diameters.

Thereafter, as shown in Fig. 9, the burden and the width of the remaining quadrangles in the cut were calculated, for both empty hole diameters. In Fig. 9, numbers from 1 to 9 are capsule numbers with a delay of 30 ms.

As can be seen from Fig. 9, the side length of the last section is obtained as 1.34 and 2.16 m for both empty hole diameters considered. As a result, it can be said that the cut is completed with 5 quadrangle for both cases. After this stage where the cut design is completed, the desired cross section should be obtained.

Since the density and relative weight strength of gelatine dynamite are 1.5 g/cm^3^ and 0.8, the bottom charge concentration is calculated from Eq. 8 for the 38 mm blast hole as in the following:

12 $${\mathrm{q}}_{\mathrm{d}}=7.854\mathrm{x}{10}^{-4}{\cdot 38}^{2}\cdot 1.5\cdot 0.8= 1.36\mathrm{ kg}/\mathrm{m}$$

The burden can then be calculated from Eq. 7 as follows:

13 $$\mathrm{B}=30\cdot 1.36/38=1.07\mathrm{ m}$$

When the burden (B), the hole depth (H) and the concentration of the bottom charge (q_b_) are known, Table 2 can be used to calculate the drilling and charging geometry of the round. The values obtained according to the calculations given in Table 2 are presented in Table 4.

**Table 4:** Drilling and charging geometry for parallel cut.

Part of the round	Burden, B (m)	Spacing, E (m)	Height of bottom charge, h_d_ (m)	Charge concentration		Stemming (m)
				Bottom, q_b_ (kg/m)	Column, q_c_ (kg/m)	
Stoping holes						
Upward and horizontally	1.07	1.18	1.0	1.4	0.68	0.5
Downwards	1.07	1.29	1.0	1.4	0.68	0.5
Roof holes	0.97	1.18	0.5	1.4	0.41	0.5
Wall holes	0.97	1.18	0.5	1.4	0.54	0.5
Floor holes	1.07	1.18	1.0	1.4	1.40	0.2

By applying the burden and spacing distances as well as stemming lengths given in Table 4 exactly, the requirements of Turkish law are violated. The aforementioned legislation states that “The height of the explosive charge cannot exceed half the hole depth. The remaining space is filled with stemming material”. If the blastholes are charged as required by the relevant legislation, the burden and spacing distances of the blastholes given in Table 4 may not work as desired or an unsuccessful blasting may be encountered.

Blasting patterns were prepared for both empty hole diameters by taking into consideration the drilling and charging geometry data given in Table 4 as preliminary information. It should be rearranged so that the height of the explosive charge does not exceed half the hole depth. While making this arrangement, the specific charge concentration was kept the same (0.286 m^3^/kg) and the geometric properties of the cross section were taken into account. In other words, rearrangement was performed keeping the charge concentration constant relative to its original value. In this case, it will be necessary to use seven cartridges of gelatine dynamite in the stoping holes and 6 cartridges in the contour holes. The floor holes will be charged half-length of the blasthole as described earlier. Plastic water cartridges were used to stem the uncharged parts of the blastholes.

**Figure 10:**
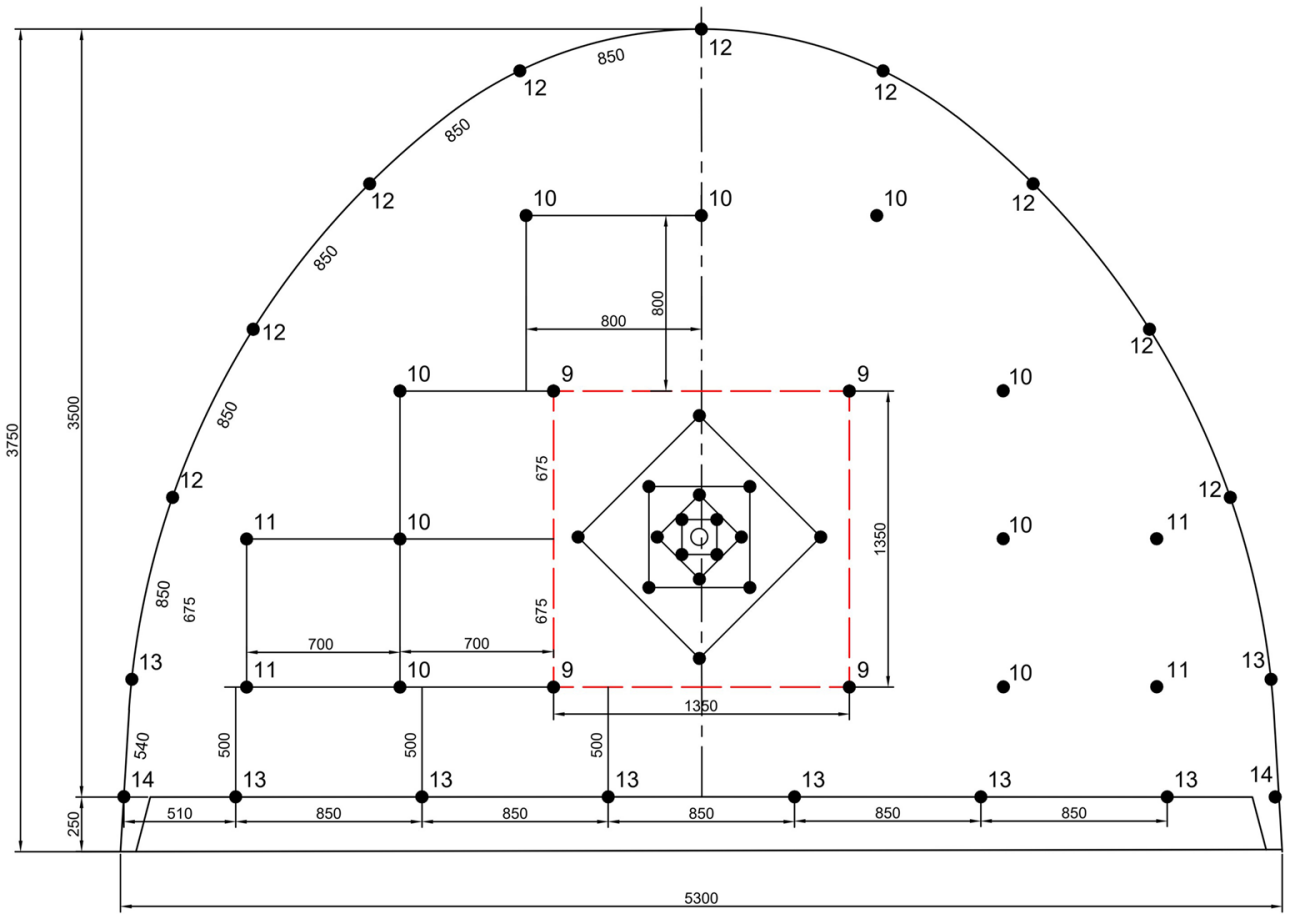
The arrangement of blastholes and firing sequences in excavation of 14 m^2^ cross-section development roadway for 76 mm empty hole diameter (see Fig. 9 for cut details).

**Figure 11:**
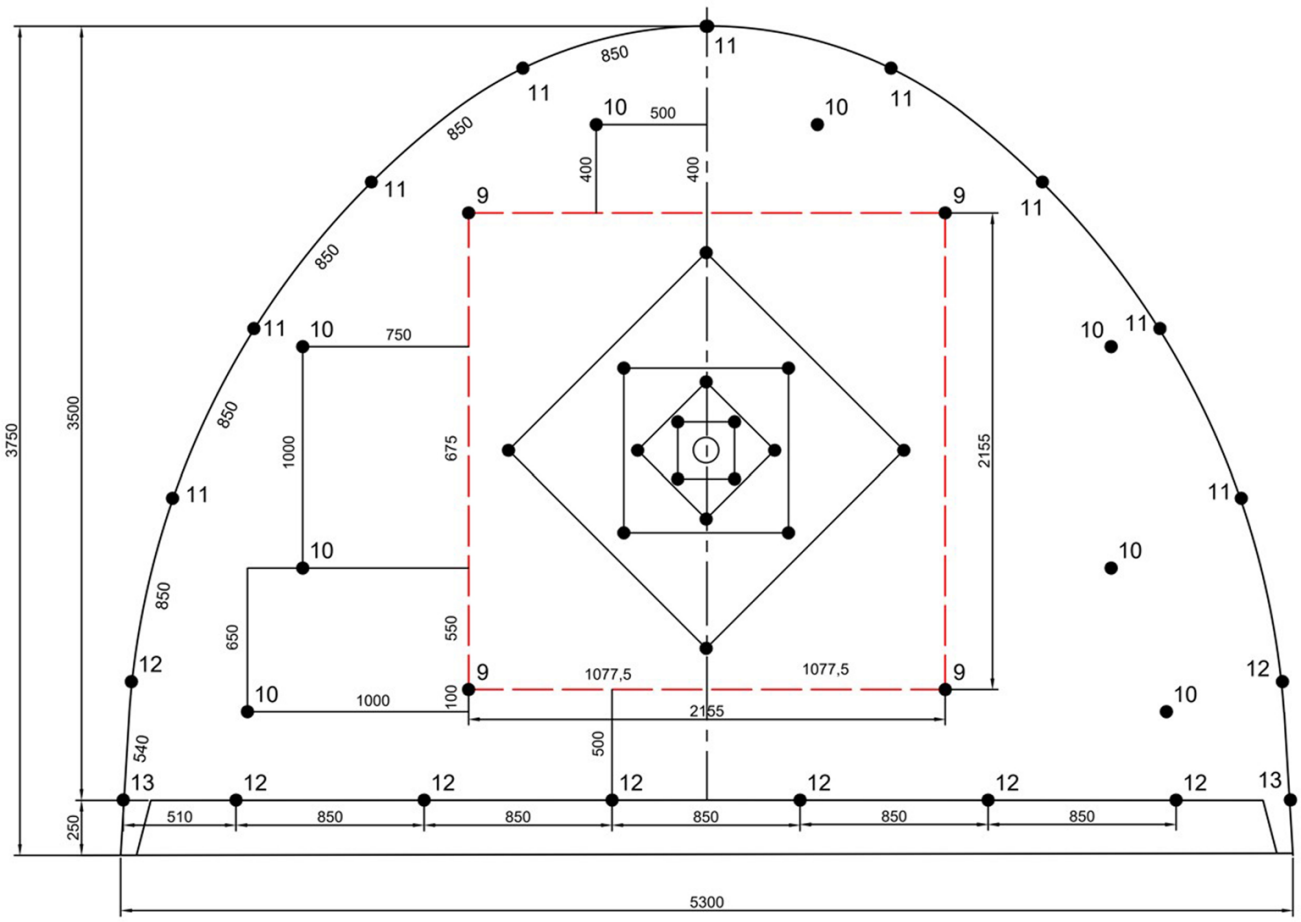
The arrangement of blastholes and firing sequences in excavation of 14 m^2^ cross-section development roadway for 115 mm empty hole diameter (see Fig. 9 for cut details).

In brief, the burden and spacing distances are adjusted to ensure that the specific charge concentration is the same as the value obtained in the calculations if the blasthole is charged to half the hole depth. The arrangement of blastholes and firing sequences in excavation of 14 m^2^ cross-section development roadways for 76 mm and 115 mm empty hole diameters is presented in Figs. 10 and 11, respectively. In these figures, the numbers on the blast holes show the firing sequence (delay order). All distances are in mm as well. As can be seen, delay numbers up to 14 were used in blast rounds considering the available millisecond and half-second capsules. The cut details were provided earlier.

The contour holes (roof holes, wall holes and floor holes) must be angled out of the contour so that the tunnel can be opened with its designed cross-sectional area. There is an angle between the practical (drilled) tunnel profile and the theoretical tunnel profile called the look-out angle. In a tunnel drilled parallel to its theoretical line, the face gets smaller and smaller after each round. It is recommended that the look-out does not exceed 10 cm + 3 cm/m hole depth^10^. Look-out is about 19 cm in this case.

### V-cut designs (gateroads and drifts)

The mine uses methane safe permissible dynamite to create gateroads (headgates and tailgates) and drifts because the electro-hydraulic drill is inefficient in such short and narrow galleries.

At the mine, blasting is done in three stages; first the cut holes are blasted, then stoping holes are drilled and blasted. In another shift, the remaining holes in the roadway face (contour holes) are drilled and blasted. 1 m of advance is provided for 12.5 m^2^ or 10 m^2^ cross-sections with 60–90 blastholes.

In this section, two different V-cut designs were made for 12.5 m^2^ cross-section with the targeted advance being 1.3 m and 2.4 m by using single and double V-cut designs. Drills of 1.6 m were included in the single V design and 2.4 m in the double V design. Both drills used at the mine have a diameter of 32 mm.

The average hole depth can be drilled by a 1.6 m drill with 95% efficiency is H = 1.5 m. To provide the 60° peak angle of cut holes, theoretical advance is A = H.Cos30^o^ = 1.3 m. In a similar manner, the average hole depth can be drilled by a 2.4 m drill with 95% efficiency is H = 2.3 m. To provide the 60° peak angle, theoretical advance is A = H.Cos30^o^ = 2.0 m.

For the density of methane safe dynamite 1.1 g/cm^3^ and the relative weight strength of methane safe dynamite S = 0.56, the bottom charge concentration q_b_ is calculated from Eq. (8) as 0.495 kg/m for the 32 mm blast hole.

The burden correction coefficient for angle of cut holes α_v_ = 60° is f = 1. The burden B can then be calculated from Eq. (9) as 0.46 m.

Since the spacing (E) for the cut holes should be 0.8 times the burden (B), the spacing for the cut holes is E = 0.46 × 0.8 = 0.37 m. When the burden (B) and the concentration of the bottom charge (q_b_) are known, Table 2 can be used to calculate the drilling and charging geometry of the round. The values obtained according to the calculations given in Table 2 are presented in Table 5 for the single V cut design.

**Table 5:** Drilling and charging geometry for single V cut.

	Cut	Stoping	Wall	Roof	Floor
Bottom charge concentration q_b_, kg/m	0.495				
Burden B, m	0.46		0.42		0.46
Spacing E, m	0.37	0.51	0.46		0.51
Bottom charge length h_d_, m	0.50		0.40	0.3	0.50
Column charge concentration q_c_, kg/m	0.25	0.2		0.15	0.50
Stemming h_s_, m	0.14	0.23	0.21		0.10
Column charge length h_k_,m	0.86	0.77	0.92	1.04	0.90

According to this calculation, it will be necessary to use four cartridges of methane safe dynamite in the cut holes. This is followed by three cartridges in the stoping and wall holes, two cartridges in the roof holes and six cartridges in the floor holes. In this case, the cut and floor holes will, however, contravene relevant legislation. As a consequence, just like in parallel hole cut design, the amount of dynamite must be rearranged so that the explosive charge does not exceed half the depth of the blasthole.

**Figure 12:**
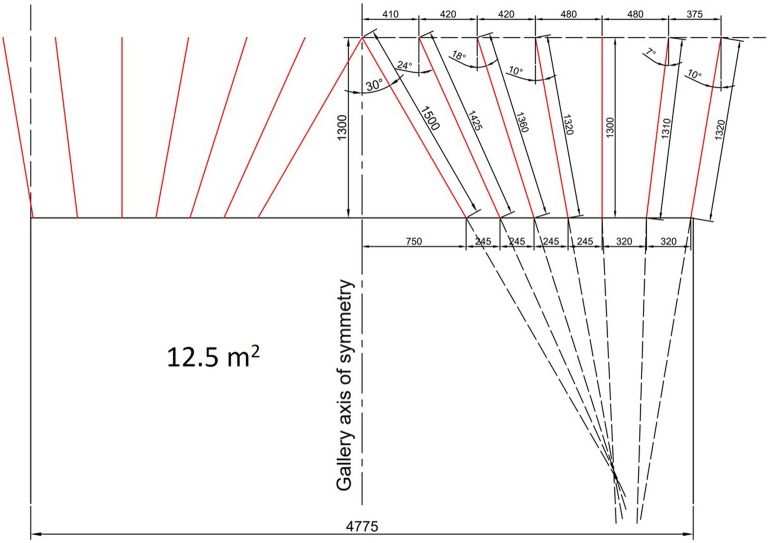
The arrangement of single V cut holes.

**Figure 13:**
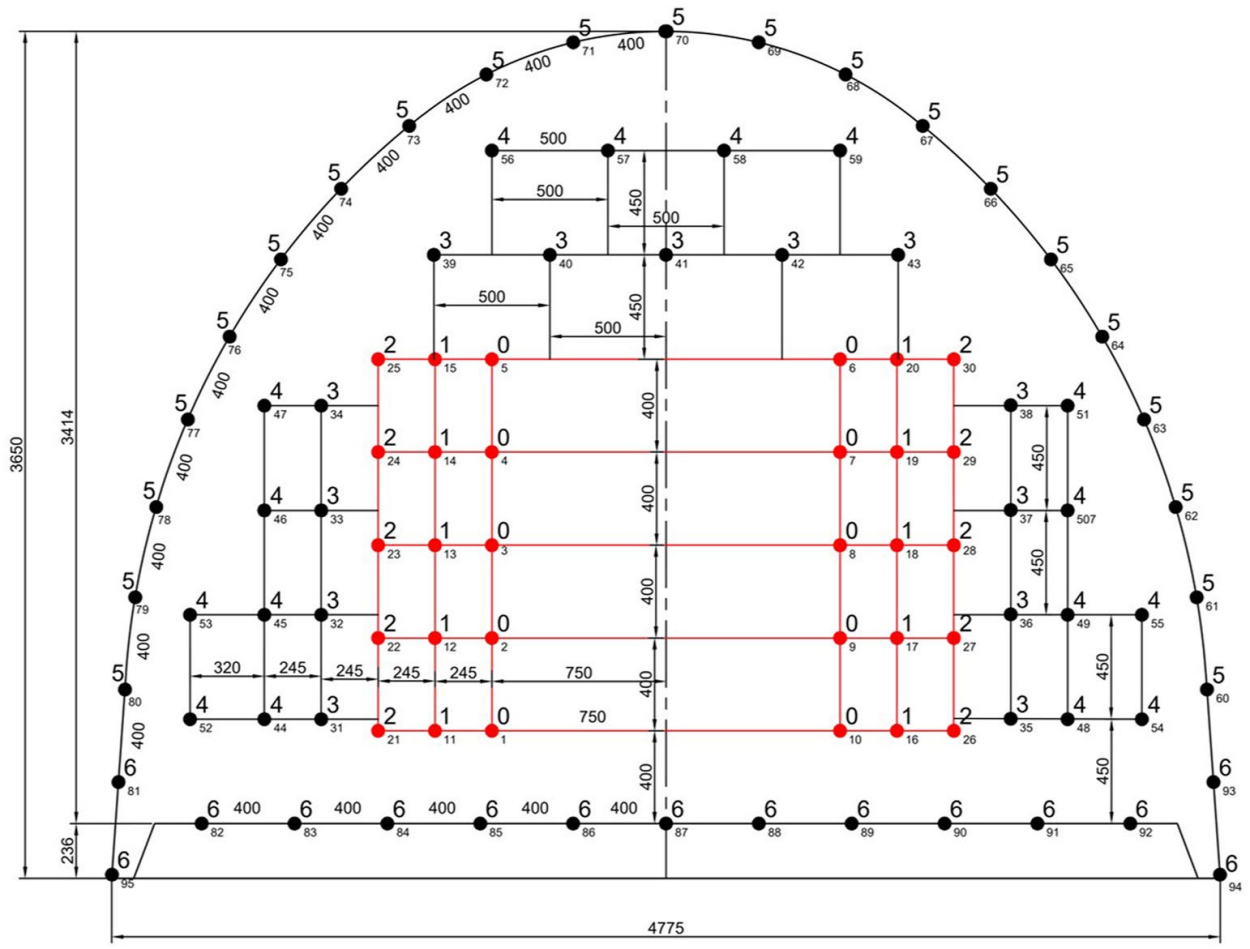
The arrangement of blastholes and firing sequences in excavation of 12.5 m^2^ cross-section for a single V cut pattern.

The arrangement of single V-cut holes is shown in Fig. 12 and the arrangement of blastholes and firing sequences in excavation of 12.5 m^2^ cross-section galleries for single V-cut pattern is presented in Fig. 13. In Fig. 13, numbers on the blast holes from 0 to 6 are capsule numbers with a delay of 30 ms. In the rearranged design pattern, it is recommended to use three cartridges of methane safe dynamite in the cut, stopping and wall holes, two cartridges in the roof holes and three cartridges in the floor holes.

In Table 6, a summary of the classic single V-cut pattern that will provide 1.3 m advance for 12.5 m^2^ cross-section is presented. In order to minimize the amount of available millisecond and half-second capsules that must be used, the cut height was chosen as long as possible. However, the firing sequences given here are still valid for galleries driven into rock. Due to the fact that delayed firing is prohibited on gateroads, each blasthole group such as cut, stoping and perimeter must be detonated individually in accordance with relevant regulations. In this case, initially, the cut holes numbered 0, 1, and 2 will be drilled and blasted. Then, after the cut cavity is formed, the stopping holes numbered 3 and 4 will be drilled and blasted. Finally, the contour and floor holes numbered 5 and 6 will be drilled and blasted to give the desired cross-section to the gateroads.

**Table 6:** Summary of blast pattern with single V-cut for 1.3 m advance at 12.5 m^2^ cross-section.

Hole no.	Hole number	Inclination	Delay no.	Dynamite per hole	Total dynamite
1–10	10	30° inward	0	3	30
11–20	10	24° inward	1	3	30
21–30	10	18° inward	2	3	30
31–38	8	10° inward	3	3	24
39–43	5	Straight	3	3	15
44–51	8	Straight	4	3	24
52–55	4	10° outward	4	3	12
56–59	4	Straight	4	3	12
60–80	21	10° outward	5	2	42
81–95	15	10° outward	6	3	45
Total	95				264 = 33 kg

This type of ignition also has its own negative effects. As an example, while drilling and blasting (charging and ignition) of blastholes can be done in one step, they are done in three stages as mentioned above. Further, the simultaneous firing of three rows of cut holes increases rock fixation and makes it more difficult to break and evacuate rocks from the face.

By using a 1.6 m drill, it is possible to provide 1.3 m advance in theory with the classic single V-cut blasting pattern. In order to make more advance, it is necessary to switch to the double V-cut pattern. To achieve this, a drill of 2.4 m must be used. With the use of a drill of this size, it will theoretically be possible to provide 2.0 m of advance. Using the chart in Fig. 8, the height of the cut (C) and the burdens B_1_ and B_2_ for the cut are determined to be 1.35, 0.950 and 0.725, respectively, for a linear charge concentration of methane safe dynamite of 0.625 kg/m.

According to this calculation, it will be necessary to use six cartridges of methane safe dynamite in the cut holes, stopping holes and wall holes, five cartridges in the roof holes and seven cartridges in the floor holes. As previously mentioned, the cut and floor holes will again contravene relevant legislation. Under these conditions, the amount of dynamite should be rearranged so that the height of the explosive charge does not exceed half the hole depth. This is done by bringing the holes closer together, as previously done in the parallel hole cut.

**Figure 14:**
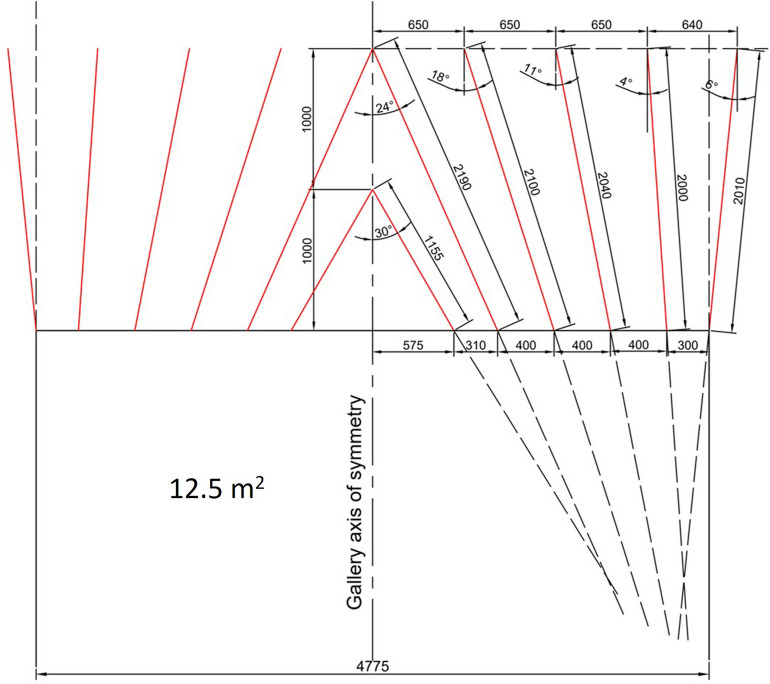
The arrangement of double V cut holes.

**Figure 15:**
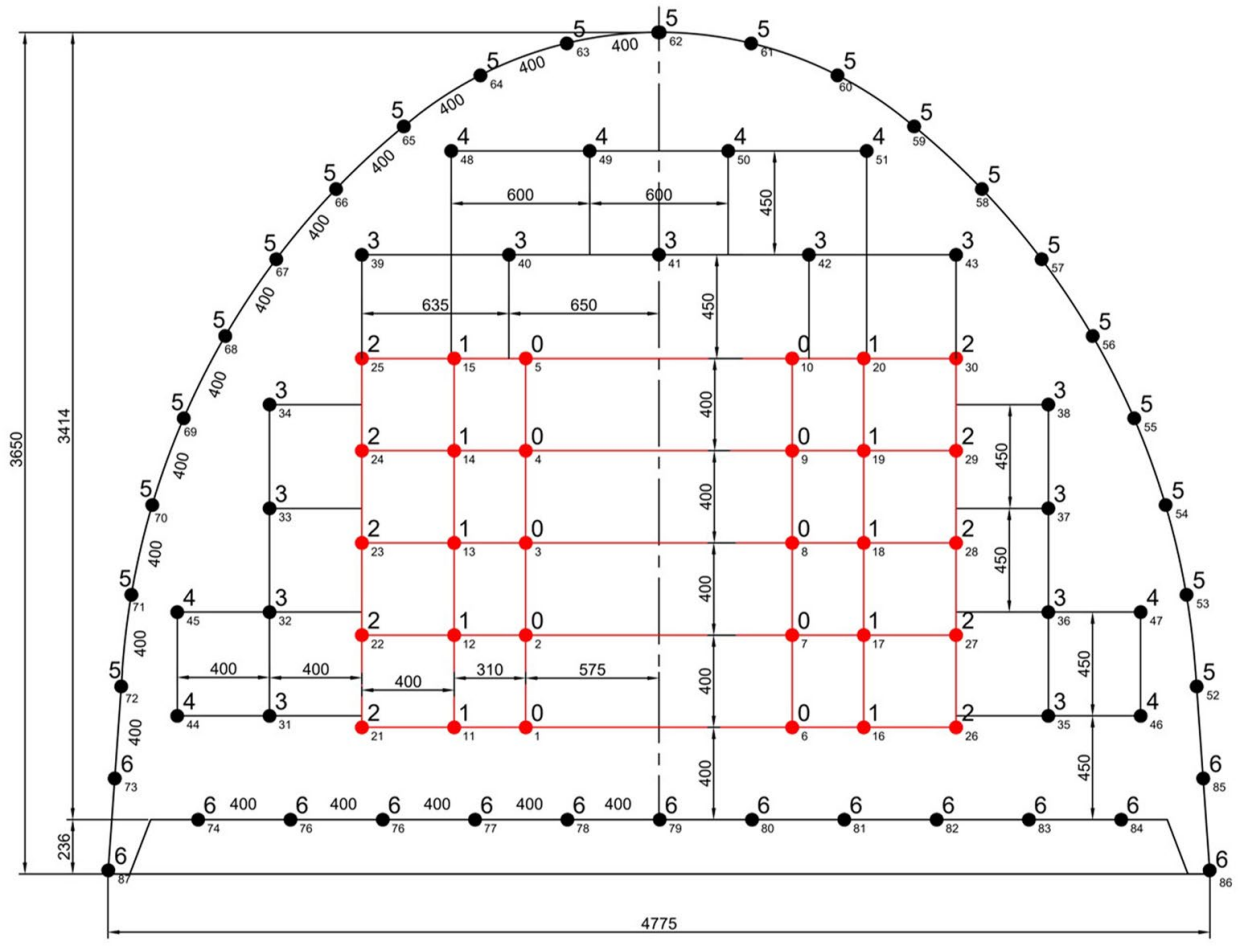
The arrangement of blastholes and firing sequences in excavation of 12.5 m^2^ cross-section for a double V cut pattern.

The arrangement of double V-cut holes is shown in Fig. 14 and the arrangement of blastholes and firing sequences in excavation of 12.5 m^2^ cross-section galleries for double V-cut pattern is presented in Fig. 15.

In Table 7, a summary of the classic double V-cut pattern that will provide 2.0 m advance for 12.5 m^2^ cross-section is presented.

**Table 7:** Summary of blast pattern with double V-cut for 2.0 m advance at 12.5 m^2^ cross-section.

Hole no.	Hole number	Inclination	Delay no.	Dynamite per hole	Total dynamite
1–10	10	30° inward	0	2	20
11–20	10	24° inward	1	5	50
21–30	10	18° inward	2	5	50
31–38	8	11° inward	3	4	32
39–43	5	straight	3	4	20
44–47	4	4° inward	4	4	16
48–51	4	straight	4	4	16
52–72	21	6° outward	5	3	63
73–87	15	6° outward	6	5	75
Total	87				342 = 43 kg

### Sample solutions for gateroads with coal seam

**Figure 16:**
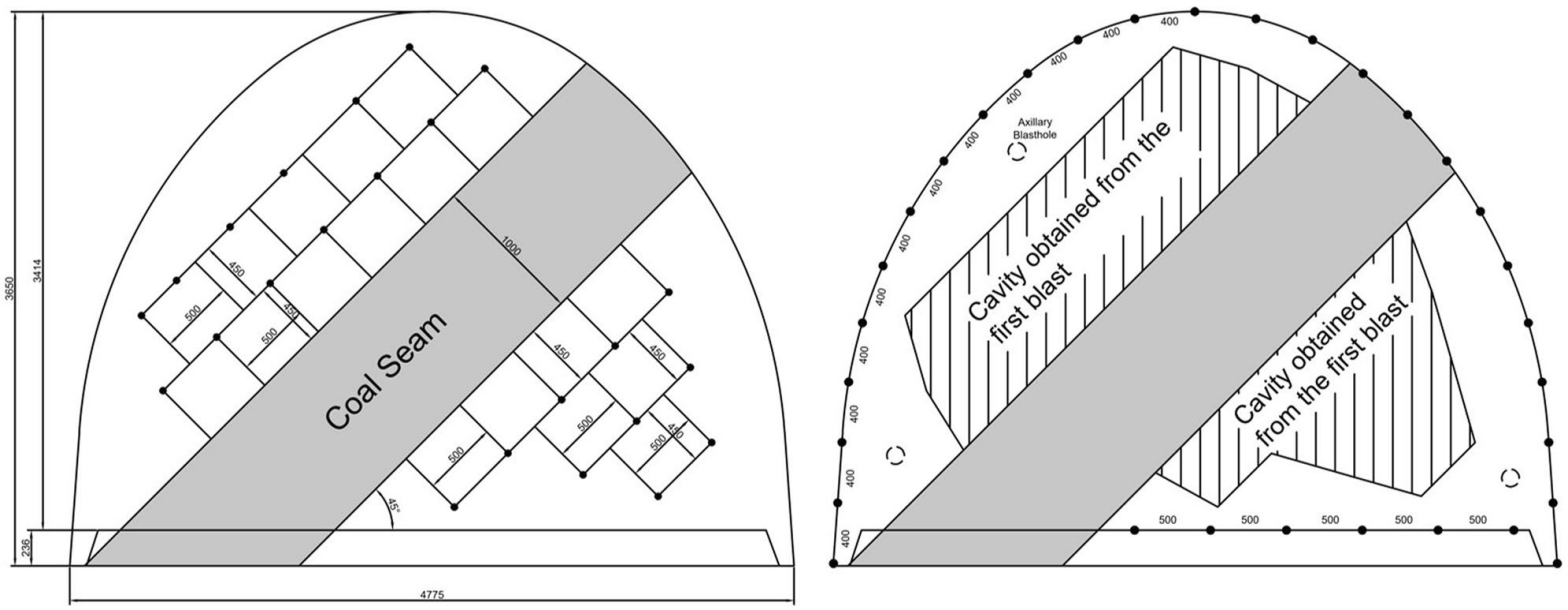
Selected gateroad geometry and placement of the first (**a**) and second (**b**) blast holes.

In the Karadon mine, coal production is carried out by Kilimli and Gelik Enterprises. Two different real cases were selected according to the conditions encountered at the mine. In the first case, a coal seam of 1 m thickness with 45° inclination was selected in the 54,516 south gallery which crosses the Kurul seam at the −460 level of Kilimli Enterprise. For the selected gateroad example, the required free surface will be created by first excavating the coal at the gateroad face. After that, as shown in Fig. 16a, the first holes to be blasted will be drilled in a line parallel to the coal seam at 45 cm burden and 50 cm spacing. To blast effectively, the rows of blastholes need to be drilled transversely to each other. It is recommended to drill straight blastholes at 2.1 m depth and then charge them with four cartridges of methane-safe dynamite, detonating them simultaneously without delay. After the first blastholes are blasted and the tallow is taken, the contour holes should be drilled at 2.15 m depth with an angle of 6° to the outward as shown in Fig. 16b. The second blastholes need to be drilled in the desired cross section, with roof and wall holes being 40 cm apart, and floor holes 50 cm apart. Roof and wall holes should be charged with three cartridges of methane safe dynamite and floor holes with four cartridges of methane safe dynamite. All of the contour holes must be blasted at the same time. Auxiliary holes may be drilled and blasted based on the blasting cavity obtained from the first blast.

In the second case, a case with a coal seam with a thickness of 1 m and an inclination of 55° with intercalations parallel to the seam was chosen in the 41,505 north gallery which crosses the Akdag seam at the −460 level of Gelik Enterprise. If the free surface is created by excavating the coal seam at the face, the first holes can be drilled perpendicularly to the face. This is similar to the first example explained previously. This time, the case of not excavating the coal seam was discussed.

**Figure 17:**
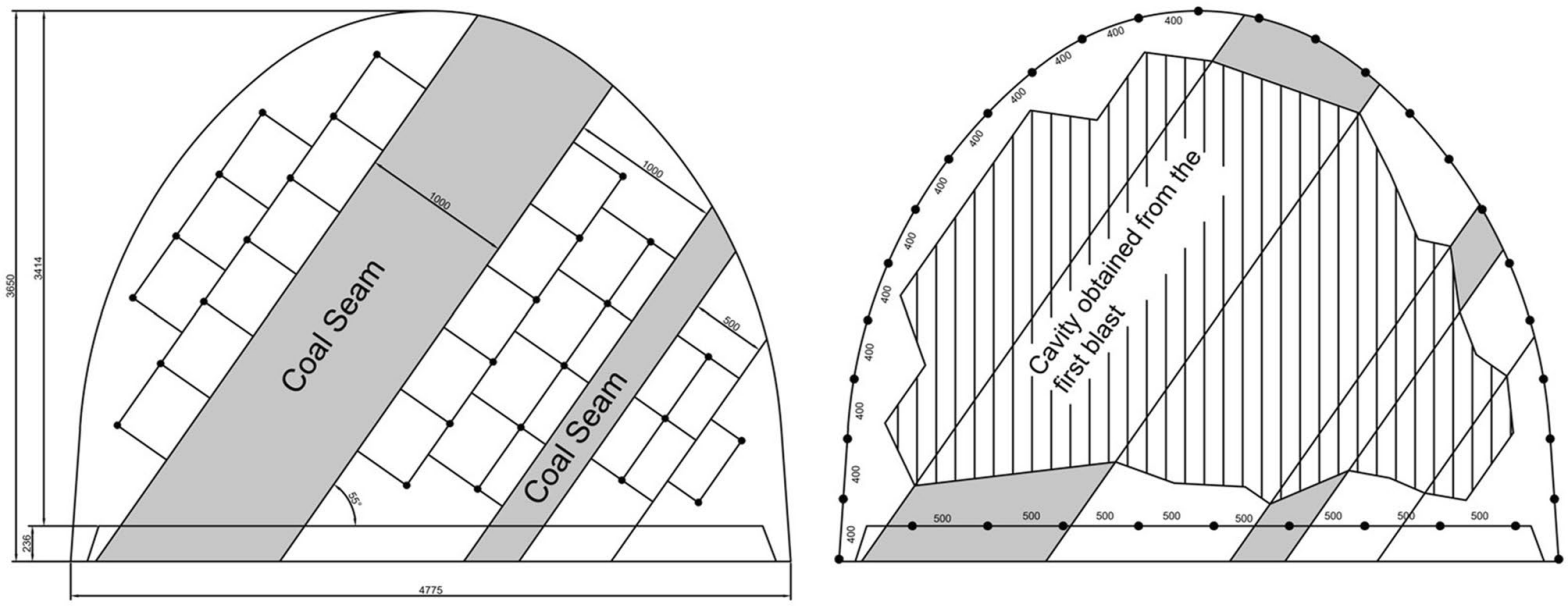
Chosen gateroad geometry and placement of the first and second blast holes.

In this case, the first holes to be blasted will be drilled along a line parallel to the coal seam at 40 cm burden and 40 cm spacing. This is shown in Fig. 17a. These first blastholes should be drilled at an 11° angle at a depth of 2.15 m inclined towards the coal seam. This inclination may be reduced in the second row of blastholes. Each blasthole was detonated at the same time without delay capsules and charging was accomplished using four cartridges of methane-safe dynamite.

After the first blastholes are blasted and the tallow is taken, the contour holes should be drilled at 2.1 m depth with an angle of 6° to the outward as shown in Fig. 17b. Second blastholes should be drilled in the desired cross section with roof and wall holes separated by 40 cm and floor holes by 50 cm. Roof and wall holes should be filled with three cartridges of methane safe dynamite and floor holes with four cartridges of methane safe dynamite. All of the contour holes must be blasted at the same time.

## Conclusions

The objective of this study is to recommend simple solutions to the blasting difficulties that are encountered in coal mines in which there are limitations arising from legal requirements. Turkish mining legislation contains some information about drilling and blasting applications in underground coal mines. However, the lack of specific design criteria in the literature for such conditions causes ineffective blast designs. In addition, rock excavation is inefficient because of this ineffective blast design.

The relevant legislation articles will be violated if blast design values are calculated according to the equations suggested in the literature for coal mine blast designs. On the other hand, if the blastholes are charged as required by the relevant legislation, the burden and spacing distances calculated from the equations given in the literature may not work as desired or even unsuccessful blasting may be encountered.

This study summarizes and analyses the blasting practices currently employed at Karadon mine along with their disadvantages. Following that, using the methods suggested in the literature, new blasting designs were made, and these designs were revised in accordance with legal requirements. Parallel hole cut designs were made for development roadways, whereas angled hole cut designs were made for gateroads and drifts. In order to comply with legal regulations, the explosive charge in each blasthole has been rearranged so that the explosive charge does not exceed half the depth of the blasthole. In this case, the burden and spacing distance of each blasthole are adjusted to achieve the same specific charge concentration obtained from the equations given in the literature. Among the information provided in this context were the drilling pattern, the amount of delayed blasting caps, and the amount of dynamites. Consequently, a shortening of the required work cycle and an increase in progress per shift have been achieved as a result of the designs implemented in the application mine. One of the key findings of this study is that rearrangement of the burden and space distances of blastholes by keeping the charge concentration constant from its original value calculated from literature, is an appropriate engineering solution for blast designs in underground coal mines. This proposed approach can be applied in similar applications.

## Data Availability

The datasets used and/or analysed during the current study available from the corresponding author on reasonable request.
